# Ionizing radiation induces senescence of dermal papilla cells through inhibiting the FOXM1–KIF20A pathway

**DOI:** 10.1016/j.gendis.2026.102126

**Published:** 2026-03-04

**Authors:** Zou Jia, Kai Tian, Yinxuan Suo, Wushuang Xu, Pingfan Wu, Wei Sun, Lijun Wu, Lei Sheng, Xiaozhong Zhou

**Affiliations:** a Department of Orthopedics, The Second Affiliated Hospital of Soochow University, Suzhou, Jiangsu 215004, China; b Department of Plastic and Aesthetic Surgery, The Second Affiliated Hospital of Soochow University, Suzhou, Jiangsu 215004, China; c Department of Burns and Plastic Surgery, Affiliated Suzhou Hospital of Nanjing Medical University, Suzhou, Jiangsu 215002, China

Radiation-induced hair follicle injury (RHFI) is a complication after radiotherapy, but lacking effective therapies. Dermal papilla cells (DPCs) are considered the command center of hair follicles.^1^ Yet, little is known about the effects of DPCs in RHFI. In this study, ionizing radiation (IR) induces cellular senescence in the dermal papilla in a mouse model. RNA sequencing analysis shows that different expression genes are enriched in the cellular senescence pathway. RNA and protein analysis reveal that IR significantly inhibits the FOXM1–KIF20A pathway, which regulates cell cycle and cellular senescence. *FOXM1* or *KIF20A* knockdown leads to inhibition of CCNB1 and CDK1, along with G2/M phase arrest and cellular senescence of DPCs. The activation of the FOXM1–KIF20A pathway alleviates G2/M phase arrest and cellular senescence post-IR. These data reveal that IR inhibits the FOXM1–KIF20A pathway, resulting in the inhibition of CCNB1 and CDK1. Then, G2/M phase arrest is promoted. Finally, cellular senescence occurs in DPCs post-IR, suppressing the ability of DPCs to induce hair follicle repair and regeneration. Our study implicates the FOXM1–KIF20A pathway as a potential target for therapies of RHFI.

Radiation therapy is a cornerstone in head and neck cancer management, yet RHFI remains a prevalent complication with limited therapeutic options.^2^ This study investigates the role of DPCs—key regulators of hair follicle cycling—in RHFI. We demonstrate that IR induces DPC senescence through G2/M phase arrest via inhibition of the FOXM1–KIF20A pathway. Restoring this pathway alleviates radiation-induced injury, offering mechanistic insights and potential therapeutic targets for RHFI.

RHFI is a well-documented adverse effect of radiation therapy for head and neck malignancies, characterized by hair loss and follicular structural damage. Clinical observations in a cranial teratoma patient revealed significant reductions in hair density and thickness within the irradiated field (Fig. S1A–S1C), mirroring preclinical findings in murine models. To recapitulate RHFI, C57BL/6 mice were exposed to electron-ray irradiation at 5Gy, 10Gy, or 15Gy on the dorsal skin. While 5Gy caused no apparent follicular injury, 10Gy and 15Gy induced progressive hair sparseness (Fig. S1E). The 15Gy group exhibited 100% mortality within two weeks, likely due to the combined effects of gastrointestinal impairment and bone marrow failure.^3^ Histopathological analysis of 10Gy-irradiated skin showed a reduction in hair follicle density and length compared with controls (Fig. 1A), accompanied by increased skin perfusion indicative of acute inflammation (Fig. S1F and S1G). By day 7 post-IR, regrown hair showed a 75% reduction in weight, 28.4% shorter length, and 42.0% thinner diameter (Fig. S1H–S1N), confirming that 10Gy irradiation reliably induces clinically relevant RHFI without lethal systemic effects.

**Figure 1:**
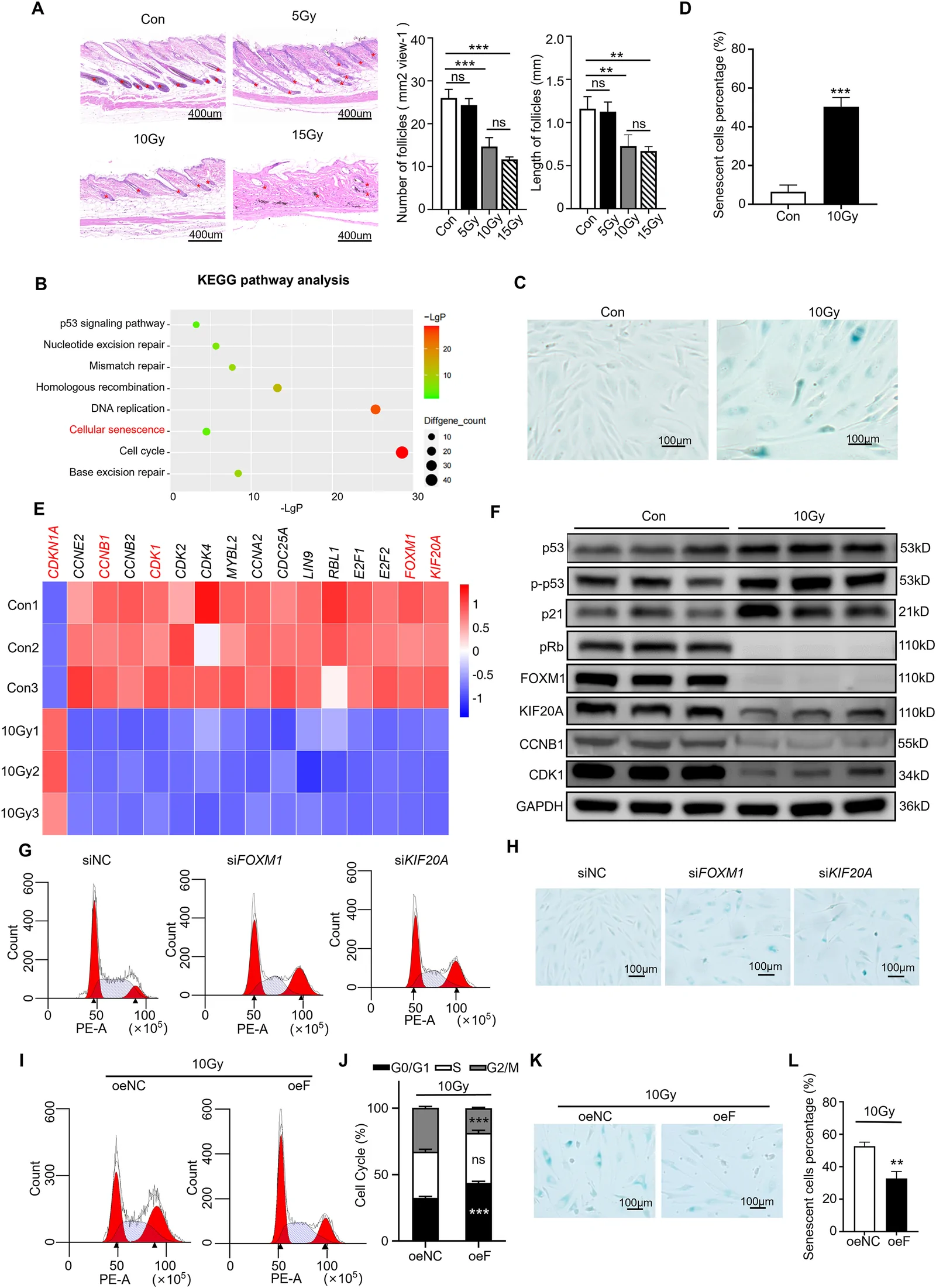
Ionizing radiation induces senescence of dermal papilla cells (DPCs) through inhibiting the FOXM1–KIF20A pathway. **(A)** Quantification of follicle number and length after 0Gy, 5Gy, 10Gy, and 15Gy irradiation at day 7 (*n* = 3). **(B)** KEGG pathway analysis of differentially expressed genes (DEGs) in DPCs 3 days after 0/10Gy irradiation. Top 8 results reserved. **(C)** Senescence-associated β-galactosidase activity (SA-β-gal) of DPCs 7 days after 0/10Gy irradiation (*n* = 3). **(E)** Heatmap representing genes of the cellular senescence pathway and FOXM1 downstream KIF20A in DEGs. **(F)** Western blotting analysis of p53, p-p53, p21, pRb, FOXM1, KIF20A, CCNB1, and CDK1 in DPCs 3 days after 0/10Gy irradiation. GAPDH was used as the loading control, and results were relative to the control group. **(G)** Cell cycle analysis of DPCs 3 days after transfection with siNC, siFOXM1, or siKIF20A. **(H)** SA-β-gal staining of DPCs 7 days after transfection with siNC, siFOXM1, or siKIF20A. **(I, J)** Cell cycle analysis of DPCs 3 days after transfection (*n* = 3). **(K, L)** SA-β-gal activity of DPCs 7 days after transfection (*n* = 3). Data were presented as mean ± standard deviation. ns: non-significant. ∗*p* < 0.05, ∗∗*p* < 0.01, and ∗∗∗*p* < 0.001.

DPCs, specialized mesenchymal cells critical for hair follicle development and cycling, were evaluated for radiation-induced dysfunction. In murine models, which were synchronized to the anagen phase at the time of irradiation (P32), the control group remained in anagen 7 days later (P39). In contrast, 10Gy irradiation induced a premature entry into the catagen/telogen phase. This pathological disruption of the hair cycle was evidenced by a significant reduction in ALP activity—a marker of DPC inductive capacity during anagen (Fig. S2A and S2B). Consequently, BrdU staining revealed a 40% decrease in proliferating matrix cells (Fig. S2C and S2D), confirming the cessation of anagen-associated proliferation and suggesting impaired epithelial–mesenchymal crosstalk. Human primary DPCs isolated from scalp biopsies were validated via α-smooth muscle actin (α-SMA) and ALP staining (Fig. S2E and S2F). Exposure to 10Gy IR reduced DPC viability to approximately 60% at 72 h (Fig. S2G), induced morphological changes (enlarged, flattened morphology; Fig. S2H) at 7 days, and slightly increased apoptosis at 72 h (Fig. S2I and S2J). Reactive oxygen species levels were elevated (Fig. S2K, S2L), correlating with reduced ALP-positive cell proportion (Fig. S2M, S2N). The secretion of key paracrine factors (KGF, IGF-1, and VEGF), which are critical for hair follicle growth, was significantly reduced (Fig. S2O). The conditioned media from irradiated DPCs impaired keratinocyte colony formation by 51.1% (Fig. S2P, S2Q), indicating the hair follicle-inductive capability of DPCs impaired by irradiation.

To characterize molecular changes in irradiated DPCs, RNA sequencing of DPCs identified 1280 differentially expressed genes, including 1007 down-regulated and 273 up-regulated genes with > 2-fold with a *P*-value < 0.05, at 72 h post-10Gy IR (Fig. S3A and S3B). Pathway analysis via Kyoto Encyclopedia of Genes and Genomes (KEGG) revealed enrichment in p53 signaling, cell cycle regulation, and cellular senescence (Fig. 1B). Senescence-associated β-galactosidase (SA-β-gal) staining confirmed an increase in senescent DPCs at day 7 post-IR (Fig. 1C and D). DNA damage foci (γ-H2AX foci) accumulated by 2.5-fold at 72 h (Fig. S3C and S3D), coinciding with cell cycle analysis showing a 25% increase in G2/M phase cells (Fig. S3E and S3F). Immunofluorescence staining revealed enhanced p16 expression in dermal papillae (Fig. S3G–S3I), further validating radiation-induced senescence.

Key differentially expressed genes in the senescence pathway included up-regulation of CDKN1A (p21) and down-regulation of FOXM1, KIF20A, CCNB1, and CDK1 (Fig. 1E). Quantitative reverse transcription-PCR confirmed mRNA reductions in FOXM1, KIF20A, CCNB1, and CDK1 (Fig. S4A). Western blotting showed activation of the p53–p21 pathway (increased p53, p-p53 Ser15, and p21) and reduced Rb phosphorylation (Fig. 1F). FOXM1, a forkhead transcription factor critical for G2/M transition, and its target KIF20A, were both transcriptionally and translationally suppressed by IR (Fig. 1F; Fig. S4B). FOXM1 and KIF20A expression in the dermal papilla area of hair follicles also decreased after irradiation *in vivo* (Fig. S4C–S4F), indicating that the FOMX1–KIF20A pathway could act as an important role in radiation-induced senescence.

SiRNA-mediated knockdown of FOXM1 or KIF20A in DPCs reduced protein levels of FOXM1, KIF20A, CCNB1, and CDK1 (Fig. S5A–S5D), mirroring IR effects. Cell viability decreased by 40%–50% (Fig. S5E), with G2/M phase arrest increasing and SA-β-gal positivity rising (Fig. 1G and H; Fig. S1F and S1G). These results indicate that FOXM1–KIF20A pathway inhibition is sufficient to induce senescence, likely through downstream regulation of CCNB1/CDK1 complexes.

To validate pathway reversibility, FOXM1 was overexpressed in irradiated DPCs via plasmid transfection. FOXM1 overexpression restored protein levels of KIF20A, CCNB1, and CDK1 (Figs. S6A, S6B, S6D, S6E), improved cell viability to 85% of controls (Fig. S6C), and rescued ALP activity (Fig. S6F and S6G). DNA damage foci were reduced (Fig. S6H and S6I), and keratinocyte colony formation improved by 40% when co-cultured with FOXM1-reconstituted DPC conditioned media (Fig. S6J and S6K). Critically, G2/M phase arrest was reduced (Fig. 1I and J), and SA-β-gal positivity decreased (Fig. 1K and L), confirming that FOXM1–KIF20A activation alleviates radiation-induced senescence.

RHFI poses significant psychosocial and clinical challenges, yet its underlying mechanisms remain poorly understood. Previous studies focused on epithelial components, neglecting the role of DPCs—the “command center” of hair growth. Our findings establish DPC senescence as a critical driver of RHFI, induced by IR-mediated inhibition of the FOXM1–KIF20A pathway.

The FOXM1–KIF20A axis regulates key cell cycle regulators (CCNB1/CDK1) to maintain G2/M transition and mitotic integrity.^4^ IR-induced DNA damage activates the p53–p21 pathway, which suppresses FOXM1 transcription and KIF20A, leading to mitotic defects and senescence. This mechanism aligns with FOXM1's roles in DNA repair and mitotic spindle assembly, explaining why its loss exacerbates radiation sensitivity.

Notably, KIF20A knockdown unexpectedly reduced CCNB1/CDK1 levels, suggesting a feedback loop or direct regulatory relationship between these proteins. While the molecular link requires further clarification, our data imply that FOXM1–KIF20A acts upstream of CCNB1/CDK1 to maintain cell cycle progression.

DPCs are specialized mesenchymal cells that are critical for hair follicle development and cycling. However, little is known about the role of DPCs in RHFI. Our study shifts the focus from epithelial components to mesenchymal components (DPCs) in RHFI. We propose that IR-induced DPC senescence is a critical upstream event that, by impairing essential epithelial–mesenchymal crosstalk (Fig. S2P and Q), exacerbates epithelial damage and prevents follicular regeneration, thus highlighting DPCs as a key therapeutic target. Activating FOXM1–KIF20A via small-molecule agonists or gene therapy could potentially preserve DPC function during radiation therapy, mitigating hair loss. We focus on first identifying the key role of the FOXM1–KIF20A pathway in DPC senescence induced by irradiation. Using a specific activator or inhibitor would be crucial for further elucidating how the FOXM1–KIF20A pathway alleviates radiation-induced G2/M phase arrest and cellular senescence of DPCs. Future studies should also validate this mechanism in animal models. This study demonstrates that IR induces DPC senescence through FOXM1–KIF20A pathway inhibition, leading to G2/M arrest and impaired follicular regeneration (Fig. S7). Targeting this axis offers a promising strategy to protect hair follicles during radiotherapy.

Our study has several limitations. First, our study did not further confirm the senescence of DPCs in human RHFI samples due to such patients often undergoing non-invasive treatments and not requiring surgical intervention, making it hard to obtain human specimens and face medical ethics challenges. Second, the specific molecular link from KIF20A to CCNB1/CDK1 needs further clarification. Third, functional validation *in vivo* is a critical step for the eventual clinical translation of this research. Further studies using specific small-molecule inhibitors or activators of the FOXM1–KIF20A pathway *in vivo* are warranted.

## CRediT authorship contribution statement

**Zou Jia:** Writing – original draft, Methodology, Formal analysis, Data curation. **Kai Tian:** Visualization, Software, Methodology. **Yinxuan Suo:** Project administration, Investigation, Formal analysis. **Wushuang Xu:** Methodology, Formal analysis, Conceptualization. **Pingfan Wu:** Software, Methodology. **Wei Sun:** Formal analysis, Data curation. **Lijun Wu:** Writing – review & editing, Supervision, Project administration, Funding acquisition. **Lei Sheng:** Writing – review & editing, Supervision. **Xiaozhong Zhou:** Writing – review & editing, Supervision, Investigation, Funding acquisition.

## Ethics declaration

The patient in this manuscript has given written informed consent to the publication of the case details. The collection and analysis of images of the patient's hair condition were approved by the Ethics Review Committee of the Second Affiliated Hospital of Soochow University, China (License No: JD-LK-2019-080-01). Animals were obtained from Hangzhou Ziyuan Experimental Animal Technology (China) Co., Ltd. All experimental procedures were approved by the Animal Protection and Experimentation Committee of Soochow University (202010A177, Suzhou, China).

## Funding

This work was supported by the 10.13039/501100001809National Natural Science Foundation of China (No. 82302831), Hospital Research Fund (the 10.13039/501100014358Second Affiliated Hospital of Soochow University, China) (No. XKTJ-HRC20210015), Suzhou Science and Technology Development Project (Jiangsu, China) (No. 2022SS43), the Special Project of “Technological Innovation” Project of CNNC Medical Industry Co. Ltd. (China) (No. ZHYLZD2021002, ZHYLTD2023001), The Project of State Key Laboratory of Radiation Medicine and Protection (Soochow University, China) (No. GZK1202244, GZK1202201), and The CNNC Elite Talent Program and Jiangsu Provincial Medical Key Discipline Cultivation Unit (China) (No. JSDW202223).

## Conflict of interests

The authors declared no competing interests.
