# Supplementary material for: Ionizing radiation induces senescence of dermal papilla cells through inhibiting the FOXM1–KIF20A pathway
Source: Genes Dis. 2026 Mar 4;14(1):102126. doi: 10.1016/j.gendis.2026.102126 (PMC13571235; doi:10.1016/j.gendis.2026.102126)
Supplement: Supplementary file 1 [file mmc1.docx]

**Materials and methods**

**Human scalp hair follicle density and thickness measurement**

Scalp detector (Chowis, Korea) was utilized to capture images for quantitative analysis of hair density and hair thickness.

**Animals and irradiation**

Animals were obtained from Hangzhou Ziyuan Experimental Animal Technology (China) Co., Ltd. All experimental procedures were approved by the Animal Protection and Experimentation Committee of Soochow University (202010A177, Suzhou, China). A total of 40 female C57BL/6 mice (4 weeks old, weighing 14-15 g) were randomly divided into Control group, 5Gy group, 10Gy group and 15Gy group. Pentobarbital (5%, 50mg/kg) was used for anesthetization. Shave back hair to induce synchronous hair follicle growth at P30. At P32, the 3.5*2cm area on the back was irradiated with 10Gy Electron from linear accelerator (BJ-6B-400C, Beijing Institute of Medical Devices, China, Energy 4Mev, Dose Rate 633MU/min) while other areas were blocked by lead plates.

**Detection of skin blood perfusion**

Mice’s back skin was scanned using a laser doppler (PeriFlux 6000, Perimed) on Day 0, Day 7 and Day 14. The color of the area with higher blood perfusion tends to be red, while the opposite is blue. The average perfusion volume of the skin was recorded within 1 minute.

**Hematoxylin and eosin staining (HE)**

The back skin was removed on Day 7, then fixed in 4% formaldehyde, paraffin-embedded and sliced into 5-μm-thick sections for HE staining. Three different fields of view were randomly selected to measure the density and depth of hair follicles.

**Immunofluorescence staining**

Cells were cultured on glass dishes and irradiated with 10Gy X-rays (RS2000 X-ray, Rad Source, USA, Energy 6MV, Dose rate 575MU/min). At specific times post-IR, the cells were washed with PBS, fixed with 4% paraformaldehyde permeabilized with 0.5% (v/v) Triton-X100 (9002-93-1, Sangon) and blocked with 5% BSA. Then cells were incubated with primary antibody against Phospho-Histone H2A.X (Ser139) (γ-H2AX) (1:1000, 2577S, CST) and alpha smooth muscle Actin (α-SMA) (1:1000, ab124964, Abcam) and the secondary antibody (A-11008, 1:2000, Invitrogen). The nuclei were incubated with DAPI (62248, Thermo Fisher). DPCs were observed by a confocal laser scanning microscope (LSM800, Zeiss). The paraffin slices were performed antigen retrieval in Tris-EDTA buffer (pH 9.0) (ab93684, Abcam), permeabilized with 0.3% Triton-X100 (9002-93-1, Sangon) and blocked with 10% goat serum (C0265, Beyotime). After incubated with primary antibodies against ALP (1:200, ab126820, Abcam) FOXM1 (1:100, 5436S, CST), KIF20A (1:200, 15911-1-AP, Proteintech) and BrdU (1:10, ab8152, Abcam) overnight, slices were washed with PBST and incubated with secondary antibodies (A-11001 or A-11004, Invitrogen). Nuclei was stained with DAPI (62248, Thermo Fisher). Images were captured with a confocal laser scanning microscope (LSM800, Zeiss).

**Isolation and culture of human dermal papilla cell**

Hair follicles were obtained from excess tissue of patients receiving hair transplantation surgeries following written informed consent. All human samples were approved by Institutional Review Board (IRB) of the Second Affiliated Hospital of Soochow University, Jiangsu, China, and all patients provided written informed consents for the collection of samples and subsequent analysis (JD-LK-2019-080-01, Suzhou, China). DPCs were dissociated and cultured as previously described^48^. Briefly, dermal papillae were micro-dissected from the bulbs and transferred to cell culture in Dulbecco's modified Eagle's medium (C11995500BT, Gibco) supplemented with 10% fetal bovine serum (002-071, CTCC) and 1% Penicillin-Streptomycin-Amphotericin B Solution (PB180121, Procell). After 1 week, DPCs were collected and plated onto cell culture dishes. Primary DPCs of 3rd-6th passage were used for experiments.

**Alkaline Phosphatase (ALP) staining**

DPCs were fixed with 4% paraformaldehyde and treated with BCIP/NBT staining working solution (C3206, Beyotime) in the dark for 1 hour. The cells were observed and captured under an ordinary optical microscope.

**Cell proliferation assay**

Cell proliferation was assayed with a cell counting kit‐8 (C0038, Beyotime). Briefly, DPCs were cultured in 96‐well plates at a density of 4 × 10^3^ cells per well and received 0/2/4/6/8/10/12Gy irradiation of X-ray (BJ-6B-400C, Beijing Institute of Medical Devices, China, Energy 6MV, Dose rate 575MU/min). After three days, cells were incubated with 10% CCK‐8 reagent for 1 hour. The absorbance was measured at 450 nm with a multilabel counter.

**Apoptosis Analysis**

DPCs were seeded in six-well plates and irradiated. Three days post-IR, the apoptosis rate was assessed by an Annexin V/PI staining kit (AP107, Multi Science). Briefly, cells were collected and incubated with Annexin-V and PI. Flow cytometry was used for analysis.

**Analysis of Reactive Oxygen Species**

H2DCF-DA (S0033S, Beyotime) was used for measurement of intracellular ROS. Briefly, DPCs were seeded in six-well plates and irradiated. 72 hours later, DPCs were collected and incubated with 5 μM H2DCF-DA. The oxidation of H2DCF was detected by flow cytometry.

**Enzyme-linked immunosorbent assay (Elisa)**

ELISA was performed following the instructions of the enzyme-linked immunosorbent assay kit to measure the concentration of KGF (E08939h, Cusabio), IGF-1 (EK1131, Multi Sciences), and VEGF (E11718h, Cusabio).

**Colony forming assay**

The immortalized human keratinocyte cell line HaCaT (CBP60331, Cobioer, China) was used for co-culture experiments. The viability of keratinocyte cells (passages 5-10) was tested by trypan blue staining. Keratinocyte cells were co-cultured with 10% supernatant of DPCs collected 72 hours post-IR. After 2 weeks, cells were fixed and stained with trypan blue. The colony numbers were counted by ImageJ.

**Transcriptome analysis by RNA-seq**

RNA samples were collected from DPCs in vitro at 72 hours post-10 Gy irradiation and detected by Genmap, Biotechnology Co, Ltd. (China) for mRNA deep sequencing on an Illumina novaseq 6000 sequencing platform with 6GB reads (Illumina, CA). Total RNA was extracted from samples using a miRNeasy Micro Kit (Qiagen, Germany) following the manufacturer’s instructions. The RNA concentrations were measured using a NanoDrop 2000 spectrophotometer (Thermo, USA) at 260/280 nm and by subjecting the RNA to Bioanalyzer 4200 (Agilent, USA). The NGS libraries were prepared using VAHTS mRNA-seq v2 Libr ary Prep Kit for Illumina® (Vazyme, China). Briefly, adapters were ligated at 3’ and 5’ ends, and mRNAs were subjected to PCR for amplification. The PCR products were further purified and used for sequencing. Genes that were upregulated or downregulated >2 folds with a P-value < 0.05 were considered significantly differentially expressed. The GO analysis on differentially expressed genes was performed with an R package: cluster profiler using a P<0.05 to define statistically enriched GO categories. Pathway analysis was used to determine the significant pathway of the differential genes according to Kyoto Encyclopedia of Genes and Genomes Database (http://www.genome.jp/kegg/).

**Quantitative real-time PCR (qRT-PCR)**

Total RNA was extracted from DPCs with TRIzol (15596026, Invitrogen). RNA was reverse transcribed using HiScript II Reverse Transcriptase (R201-01, Vazyme). RT-PCR was performed with ChamQ SYBR qPCR Master Mix (Q311-02/03, Vazyme). The primers were listed in **Supplementary Tab. 1.**

**Senescence-associated β-galactosidase (SA-β-gal) activity**

7 days post-IR, SA–β-gal staining was performed using a staining kit (C0602, Beyotime). Briefly, DPCs were fixed with 4% paraformaldehyde and incubated with SA-β-gal solution at 37 °C without CO_2_ for 24h. In mice, 5mm skin frozen sections were fixed with 0.5% glutaraldehyde and incubated with SA-β-gal staining solution at 37℃ for 24h. The nucleus was stained with hematoxylin. Images were captured with a light microscope.

**Cell cycle assay**

DPCs were collected and fixed with 70% ethanol overnight at -20℃ and stained with the Cell Cycle Kit (CCS012, Multi Sciences). Cell cycle analysis was performed using a flow cytometer (Beckman, USA).

**Western blot analysis**

Proteins were extracted using RIPA butter (P0013B, Beyotime). Protein concentration was calculated with a BCA analysis Kit (P0012, Beyotime). Proteins were separated by SDS-PAGE. Primary antibodies included p53 (1:1000, ab26, Abcam), p-p53 (1:1000, ab32132, Abcam), p21 (1:500, sc-6246, Santa Cruz), pRb (1:1000, 8516S, CST), FOXM1 (1:1000, 5436S, CST), KIF20A (1:1000, 15911-1-AP, Proteintech), Cyclin B1 (1:1000, 12231S,CST), CDK1 (1:1000, 9116S, Cell Signaling Technology), GAPDH (1:50000, 60004-1-Ig, Proteintech) and Beta-Actin (1:50000, 66009-1-Ig, Proteintech). Secondary antibodies were goat anti-rabbit IgG and goat anti-mouse IgG (1:1000, A0192 and A0208, Beyotime). Immunolabeling was activated by an Omni-ECL™ Femto Light Chemiluminescence Kit (SQ201, Epizyme) and captured by a GeneGnome XRQ analyzer (Syngen, UK). ImageJ was used to analyze the gray value normalized to GAPDH or Beta-Actin.

**Cell transfection**

The siRNAs to deplete FOXM1 or KIF20A and plasmid to overexpress FOXM1 were purchased from GenePharma (China). Details are listed in **Supplementary Table 1.** Lipofectamine 3000 (L3000001, Invitrogen) was used for transfection.

**Supplementary Table 1.**

| siRNA | Sense（5’-3’） | Anti-Sense（5’-3’） |
| --- | --- | --- |
| FOXM1 siRNA-461 | GCUGGGAUCAAGAUUAUUATT | UAAUAAUCUUGAUCCCAGCTT |
| FOXM1 siRNA-1223 | GCCAACCGCUACUUGACAUTT | AUGUCAAGUAGCGGUUGGCTT |
| FOXM1 siRNA-1407 | GCUCAUACCUGGUACCUAUTT | AUAGGUACCAGGUAUGAGCTT |
| KIF20A siRNA-1958 | GCAUCUACCUAUGAUGAAATT | UUUCAUCAUAGGUAGAUGCTT |
| KIF20A siRNA-2823 | CCACUUGUGAUGACAUCUUTT | AAGAUGUCAUCACAAGUGGTT |
| KIF20A siRNA-3068 | CCAACCUGCCAAAGCUCAATT | UUGAGCUUUGGCAGGUUGGTT |

Supplementary figures and legends:

**Figure S1. Radiation induces hair follicle injury. (A-C)** Hair density and thickness in the normal area and irradiated area of a cranial teratoma patient (n=3). **(D)** Animal irradiation process. PD is the abbreviation for “Postpartum Day” and PW for “Postpartum Week”. **(E)** General condition of hair growth after 0Gy, 5Gy, 10Gy and 15Gy irradiation at Day0, Day7 and Day14. **(F-G)** Skin Perfusion of mice back at Day7 and Day15 (n=3). **(H-I)** Weight of newly grown hair in the 3.5cm * 2.5cm area on the back at Day7 (n=3). (**J-M)** Length and thickness of newly grown hairs at Day7 (n=15). Data are presented as the mean ± SD. ns: non-significant. *p < 0.05, **p < 0.01, ***p < 0.001.

**Figure S2. Radiation decreases the activity of the DPCs. (A-B)** Fluorescence staining analysis of ALP expression in hair follicles 7 Days post-IR. Nuclei were stained with DAPI. DPs were labeled with dotted lines. Bar: 25um. **(C-D)** Cell proliferation analysis on hair follicles 7 Days post-IR through BrdU staining. Nuclei were stained with DAPI. DP were labeled with dotted lines. Bar: 25um. **(E-F)** Cell identification of dermal papilla cells using α-SMA Fluorescence staining and ALP staining. **(G)** Relative cell viability of DPCs after 0, 2, 4, 6, 8, 10 and 12Gy irradiation 3 Days post-IR. **(H)** Cell shape of DPCs 3 Days post-IR. **(I-J)** Apoptosis analysis of DPCs 3 Days post-IR. **(K-L)** Reactive oxygen species (ROS) analysis of DPCs 3 Days post-IR. **(M-N)** ALP staining of DPCs and analysis of ALP^+^ cells percentages 3 Days post-IR. **(O)** Elisa analysis of KGF, IGF-1 and VEGF in DPCs’ supernatant 3 Days post-IR. **(P-Q)** Colony formation of keratinocytes cultured with 10% supernatant of DPCs without/with 10Gy-IR. Data are presented as the mean ± SD (N=3). ns: non-significant. *p < 0.05, **p < 0.01, ***p < 0.001.

**Figure S3. Radiation induces DNA damage and cell cycle arrest in DPCs. (A)** Heatmap of the RNA-sequencing data showing DEGs in DPCs 3 days after 0/10Gy irradiation. **(B)** Volcano plot of DEGs between DPCs 3 days after 0/10Gy irradiation. **(C-D)** Fluorescence staining analysis of γ-H2AX of DPCs 3 days after 0/10Gy irradiation (n=6). Nuclei were stained with DAPI. **(E-F)** Cell cycle analysis of DPCs 3 days after 0/10Gy irradiation (n=3). **(G)** SA-β-gal activity of mice hair follicles 7 days after 0/10Gy irradiation (n=3). DP showed SA-β-gal positive staining. DPs were labeled with dotted lines. **(H-I)** p16 staining of mice hair follicles 7 days after 0/10Gy irradiation (n=3). DP showed evident p16 positive staining. DPs were labeled with dotted lines. Data are presented as the mean ± SD. ns: non-significant. *p < 0.05, **p < 0.01, ***p < 0.001.

**Figure S4. Radiation inhibits the FOXM1-KIF20A pathway in DPCs. (A)** qRT-PCR analysis of cellular senescence pathway key genes, including p53, CDKN1A, E2F1, FOXM1, CCNB1, CDK1 and FOXM1 downstream KIF20A. **(B)** Western blot analysis of p53, p-p53, p21, pRb, FOXM1, KIF20A, CCNB1 and CDK1 in DPCs 3 days after 0/10Gy irradiation. GAPDH was used as the loading control and results were relative to the control group. **(C)** Fluorescence staining analysis of FOXM1 expression in hair follicles 7 Days post-IR. **(D)** Fluorescence staining analysis of KIF20A expression in hair follicles 7 Days post-IR. Nuclei were stained with DAPI. DPs were labeled with dotted lines. Bar: 25um. Data are presented as the mean ± SD (n=3). ns: non-significant. *p < 0.05, **p < 0.01, ***p < 0.001.

**Figure S5. *FOXM1/KIF20A* knockdown promotes G2/M phase arrest and cellular senescence of DPCs. (A-B)** Western blot analysis of FOXM1, KIF20A, CDK1 and CCNB1 transfected with siNC or siFOXM1 for 2 days. GAPDH and β-actin were used as the loading control and results were relative to siNC group. Using siFOXM1-1407 for subsequent experiments. **(C-D)** Western blot analysis of KIF20A, CDK1 and CCNB1 transfected with siNC or siKIF20A for 2 days. β-actin was used as the loading control and results were relative to siNC group. Using siKIF20A-1958 for subsequent experiments. **(E)** Relative cell viability of DPCs using CCK8 3 days after transfection. **(F)** Cell cycle analysis of DPCs 3 days after transfection with siNC, siFOXM1 and siKIF20A. **(G)** Senescent DPCs percentage 7 days after transfection with siNC, siFOXM1 and siKIF20A. Data are presented as the mean ± SD (n=3). ns: non-significant. *p < 0.05, **p < 0.01, ***p < 0.001.

**Figure S6. Activation of FOXM1-KIF20A pathway alleviated DNA damage and improved hair follicle-inductive capability of DPCs. (A-B)** Transfection of different concentration oeFOXM1. Western blot analysis of FOXM1 and KIF20A two days after transfection (n=3). GAPDH was used as the loading control and results were relative to oeNC group. Using 1.5ug concentration oeFOXM1 for subsequent experiments. **(C)** Relative cell viability of DPCs using CCK8 3days after transfection (n=3). **(D-E)** Western blot analysis of CCNB1 and CDK1 two days after transfection (n=3). β-actin was used as the loading control and results were relative to oeNC group. **(F-G)** ALP staining of DPCs and analysis of ALP^+^ cells percentages 3 days after transfection (n=3). **(H-I)** Fluorescence staining analysis of γ-H2AX of DPCs 3 days after transfection (n=6). Nuclei were stained with DAPI. **(J-K)** Colony formation of keratinocytes cultured with 10% supernatant of DPCs transfected with oeNC and oeFOXM1. Data are presented as the mean ± SD. ns: non-significant. *p < 0.05, **p < 0.01, ***p < 0.001.

**Figure S7. Schematic illustration showing the possible mechanism by which IR induces cellular senescence of DPCs.** IR caused inhibition of FOXM1-KIF20A axis, suppressing the downstream CCNB1 and CDK1. Subsequently, DPCs underwent G2/M phase arrest and cellular senescence. The ability of senescent DPCs to induce hair follicles growth decreased, leading to hair follicle atrophy and loss.
